# Cost-effectiveness Analysis of Lorlatinib in Patients Previously Treated with Anaplastic Lymphoma Kinase Inhibitors for Non-small Cell Lung Cancer in Greece

**DOI:** 10.36469/jheor.2022.32983

**Published:** 2022-02-17

**Authors:** George Gourzoulidis, Oresteia Zisimopoulou, Nadia Boubouchairopoulou, Christina Michailidi, Chrissy Lowry, Charalampos Tzanetakos, Georgia Kourlaba

**Affiliations:** 1 ECONCARE LP; 2 Pfizer Hellas; 3 BresMed

**Keywords:** cost-effectiveness, lorlatinib, anaplastic lymphoma kinase, Greece, non-small cell lung cancer

## Abstract

**Background:** Non-small cell lung cancer (NSCLC), which accounts for about 80%-85% of lungcancer cases, is a leading cause of cancer-related death worldwide. Lorlatinib is a potent third-generation anaplastic lymphoma kinase (ALK) inhibitor approved for the treatment of patients with advanced, ALK-positive NSCLC previously treated with at least one second-generation ALK tyrosine kinase inhibitor.

**Objective:** The present study assessed the cost-effectiveness of lorlatinib vs pemetrexed with platinum combination of carboplatin or cisplatin (P-ChT) in Greece.

**Methods:** A partitioned survival model with three health states, referring to pre-progression, progressed disease, and death, was locally adapted from a Greek payer perspective over a lifetime horizon. Clinical and safety data and utility values applied in the model were extracted from the literature. A matching-adjusted indirect comparison of lorlatinib and P-ChT was performed. Only direct medical costs (€) from 2020 were included in the analysis. Primary outcomes were patient life years (LYs), quality-adjusted life years (QALYs), total costs, and incremental cost-effectiveness ratios per QALY and LY gained. All future outcomes were discounted at 3.5% per annum. A probabilistic sensitivity analysis was conducted to account for model uncertainty.

**Results:** The analysis showed that, over a lifetime horizon, the estimated total costs of lorlatinib and P-ChT were €81 754 and €12 343, respectively. Lorlatinib was more effective than P-ChT with 2.4 and 1.5 more LYs and QALYs gained, respectively. The generated incremental cost-effectiveness ratios of lorlatinib compared with P-ChT were €28 613 per LY gained and €46 102 per QALY gained. Probabilistic sensitivity analysis confirmed the deterministic results.

**Conclusion:** The present analysis suggests that lorlatinib may be considered as a cost-effective option compared with P-ChT in Greece for the treatment of patients with advanced, ALK-positive NSCLC whose disease has progressed after at least one second-generation ALK tyrosine kinase inhibitor. In addition, this option addresses a significant unmet medical need.

## Background

Non-small cell lung cancer (NSCLC), which accounts for about 80%-85% of lung cancer cases, is a leading cause of cancer-related death worldwide.^1^ Most patients with NSCLC present with advanced, incurable disease at diagnosis, expressing bothersome symptoms that affect their quality of life, such as fatigue, dyspnea, shortness of breath, and cough.^2,3^ Anaplastic lymphoma kinase (ALK)-positive NSCLC represents a small proportion (3%-5%) of patients with advanced NSCLC.^4^

Targeted therapies have substantially improved the survival outcomes of patients with ALK-positive NSCLC. The availability of multiple targeted therapeutic options for ALK-positive NSCLC has shown to be an important factor in the therapeutic management of patients with NSCLC.^5,6^ More specifically, crizotinib was the first ALK inhibitor approved for patients with ALK-positive NSCLC.^7^ Subsequently, second-generation ALK inhibitors such as ceritinib, alectinib, and brigatinib are the currently available oral ALK inhibitors approved by the European Medicines Agency for the first-line or second-line (ie, post-crizotinib) treatment (SLT) of ALK-positive advanced NSCLC.^8–10^ As second-generation ALK inhibitors become more widely used, including in first-line treatment, there is an unmet medical need for agents that can be used in sequence after second-generation ALK inhibitors. However, there are no targeted therapies available currently in Greece after progression on second-generation ALK inhibitors. The current standard of care remains chemotherapy, which has poor efficacy in the second-line setting and beyond, including poor penetration in the central nervous system (CNS), a common and often refractory site of metastasis for patients with ALK-positive NSCLC.^11,12^ Therefore, an alternative to chemotherapy, with broad mutation coverage and CNS penetration, is necessary to address the high unmet need for these patients.

Lorlatinib has the potential to fill this unmet medical need. Lorlatinib is a potent, brain-penetrant, third-generation inhibitor of ALK and ROS1 tyrosine kinases with broad coverage of ALK mutations. Its safety and efficacy were investigated in an open-label, multicenter, multiple-dose, dose-escalation, safety, pharmacokinetics, pharmacodynamics, and anticancer efficacy exploration study. This phase I/II clinical trial, which was specifically designed to investigate safety and efficacy in patients previously treated with an ALK inhibitor, included treatment with a second-generation ALK inhibitor. More specifically, the study included various patient expansion cohorts (EXP), based on prior therapies received and current line of treatment. The European Medicines Agency recognized the potential benefits of lorlatinib and approved it in May 2019 as monotherapy for the treatment of adult patients with ALK-positive advanced NSCLC whose disease progressed on an ALK tyrosine kinase inhibitor (TKI), thus making it currently the only approved targeted therapy after progression on second-generation ALK TKIs.^13^

Clinical trial results reported that, among patients who had received at least 1 previous ALK TKI (EXP2-5), objective response was achieved in 93 of 198 patients (47%), and objective intracranial response was observed in 51 of 81 patients (63%) with measurable CNS lesions.^14^ Objective response was achieved in 41 of 59 patients (69.5%) who had received previous crizotinib alone (EXP2-3A), 9 of 28 patients (32.1%) who had received one previous ALK TKI (not crizotinib; EXP3B), and 43 of 111 patients (38.7%) who had received 2 or more ALK TKI (EXP4-5). Among patients with measurable CNS lesions, objective intracranial response was observed in 20 of 23 patients (87.0%) in EXP2-3A, 5 of 9 patients (55.6%) in EXP3B, and 26 of 49 patients (53.1%) in EXP4 and EXP5.

Based on its efficacy data, lorlatinib appears to be an effective treatment for patients with ALK-positive, metastatic NSCLC. Nevertheless, the balance of treatment efficacy and costs should be evaluated to maximize its value for the money in healthcare spending. This need has led to the use of economic assessments comparing new treatments with existing options. The aim of the present study was to delineate the cost-effectiveness of lorlatinib compared with combination of pemetrexed with platinum-based chemotherapy (P-ChT) such as carboplatin or cisplatin for the treatment of patients with ALK-positive NSCLC previously treated with ≥1 second-generation TKI in Greece.

## Methods

A published partitioned survival model^15^ in Microsoft Excel was locally adapted to reflect the natural progression of patients with advanced, ALK-positive NSCLC previously treated with ≥1 second-generation ALK TKI. A partitioned survival model is a common modeling structure in oncology and an approach consistent with previous submissions to the National Institute for Health and Care Excellence (NICE) and published literature.^15,16^ Therefore, a partitioned survival methodology was the most suitable for this decision problem. Hence, the model evaluated the cost-effectiveness of lorlatinib compared with a combination of pemetrexed and P-ChT, such as carboplatin or cisplatin, from a public payer perspective over a lifetime horizon. The cycle length in the model was 1 month. A half-cycle correction (ie, averaging outcomes between the beginning and end of each cycle to reflect that event can occur at any point within the cycle) is applied to all costs and outcomes except for drug costs (which are incurred at the beginning of each cycle). Primary outcomes of the model were patient quality-adjusted life years (QALYs), life years (LYs), total costs per patient, and incremental cost-effectiveness ratios (ICERs) per QALY and LY gained. An annual discounting of 3.5% was applied for both effectiveness and cost estimations as this is the standard practice in these studies in Greece.^17–19^

### Cost-Effectiveness Model Description

**Figure 1.**
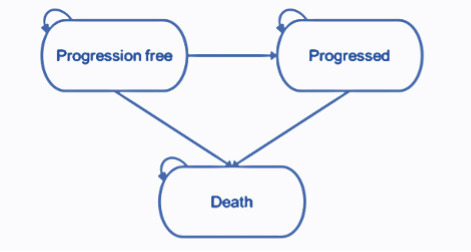
Model Structure.

The partitioned survival model structure comprises 3 health states (pre-progression, progressed disease, and death). The health states represent the key sequence of events that NSCLC patients may experience over the course of their treatment (Figure 1). All patients enter the model in the progression-free health state and remain in this state until disease progression or death. Patients who experience disease progression will transition into the progressed disease health state. In this state, the patient will move on to subsequent treatment lines before death. Subsequent treatment lines are assumed to apply only once the patient has discontinued their initial treatment where treatment is permitted to be received beyond the point of progression. Patients are not permitted to transition from the progressed disease to the progression-free health state. Transitions to the death state can occur from either the progression-free or progressed disease health states, and death is an absorbing state.

The progression-free health state is designed to capture the relatively higher quality of life while the disease is controlled prior to progression, where patients are receiving benefit from an active treatment. The progressed disease state is designed to capture the relatively poor quality of life following disease progression. The model therefore captures the changes in quality of life between pre- and post-progression.

### Target Population

The target population in the model is adult patients with ALK-positive, advanced NSCLC previously treated with ≥1 ALK TKIs. This is defined as the second-line+ treatment in the model. The patients’ characteristics used for the analysis were extracted from a phase II clinical trial.^14^

### Model Inputs

**Clinical inputs:** The clinical inputs considered in the model are overall survival (OS), progression-free survival (PFS), time on treatment, and safety. More specifically, no head-to-head clinical trials containing the treatments of interest have been reported; based on the available literature data, it was not possible to form a connected network of evidence. Therefore, to compare treatments incrementally, accounting for heterogeneity between each of the trials, a matching-adjusted indirect comparison (MAIC) was performed.^15^ MAIC and indirect comparisons have been widely used and accepted in health technology submissions to NICE, including the submissions in the ALK-positive NSCLC population.^15^

To assess the feasibility of indirect comparisons, we found that it was most appropriate to match the identified comparator studies to a subset of the lorlatinib cohorts that were most similar, particularly in terms of the line of therapy. Hence, for chemotherapy, the hazard ratio (HR) of PFS was estimated using the EXP2-3A lorlatinib cohorts, which align with the populations in the ALUR and ASCEND-5 studies.^7,20^ For these cohorts, baseline demographics and disease characteristics, including prior lines of therapy, were generally consistent with the populations in the ALUR and ASCEND-5 studies.^7,20^ Further, these estimates have been used in the recent publication of the model.^15^ Extrapolation of OS and PFS for lorlatinib was performed to estimate likely outcomes beyond the observed duration of the clinical trial. Extrapolation was undertaken by fitting standard parametric survival curves, each being fit to patient-level data from the lorlatinib trial. Parametric survival models (Weibull, log-normal, log-logistic, exponential, generalised gamma, and Gompertz distributions) were fitted to the observed OS and PFS data, and then extrapolated over the time horizon. Following visual inspection and statistical testing, the generalized gamma curve was selected for both PFS and OS in the base case analysis on the basis of model fit and plausibility of extrapolation. The PFS HR estimated from the MAIC was applied to the PFS curve for lorlatinib to obtain the comparator PFS curve. As for OS, no data were identified for chemotherapy to compare with the lorlatinib cohorts; therefore, the HR for OS was primarily assumed to be equal to the HR for PFS. This also aligns with the assumption taken by the NICE Evidence Review Group in the appraisal of crizotinib in ROS1-positive advanced NSCLC.^15^

Moreover, the MAIC could not include time on treatment within the analysis, as this was reported differently in all the studies. As such, time-on-treatment data were obtained from the clinical trial^14^ for lorlatinib, while for the P-ChT time on treatment was obtained from published studies.^7,20^ The model assumes time on treatment to be equal to PFS for lorlatinib, while for P-ChT an exponential distribution was assumed based on the median time on treatment reported in published studies.^7,20^

**Safety data:** Adverse events (AEs) of grade ≥3, occurring in >5% in any treatment arm, were included in the analysis to account for the potential cost and quality-of-life burden of experiencing events while on treatment. The incidence rates of AEs for lorlatinib were sourced from the clinical trial^14^ while those for P-ChT were obtained from published studies (**Supplementary Table 1**).^20^

**Utility weights:** Utility weights are a measure of a patient’s preference of a health state and generally range from 0 (death) to 1 (perfect health). Utility values considered in the model in the pre-progression state were obtained from the lorlatinib clinical trial^14^ for lorlatinib and Zhou et al^21^ for P-ChT. In the post-progression state, the utility values for patients who had discontinued their initial treatment were sourced from the literature.^22^ Of note, in the base case analysis, AE disutilities were excluded, with the assumption that health state utilities already capture the effect of any AEs (Table 1).

**Table 1.** Utility Values Considered in the Model.

Health State	Utility Value (Second-Line+)	Source
Progression-free		
Lorlatinib	0.785	Solomon et al^14^
P-ChT	0.747	Zhou et al^21^
Progressed disease	0.610	NICE^22^

Abbreviations: NICE, National Institute for Health and Care Excellence; P-ChT, pemetrexed plus carboplatin or cisplatin.

**Healthcare resource use and cost estimation:** Following a public payer perspective, direct country-specific costs related to monitoring costs were split into progression-free and progressed disease health state costs per cycle. End-of-life care, drug acquisition and administration, AE, and post-progression treatment costs were also considered in the analysis. Direct medical costs (in €) reflected only the year 2020.

**Pre-progression state costs:** The total drug acquisition costs were calculated by combining the drug dose of each comparator, as obtained from the relevant summary of product characteristics with the corresponding reimbursed drug cost. In addition, to better reflect real-world clinical practice, a relative dosing intensity value was applied to all patients in the model; the underlying assumption is that not all patients who are receiving treatment necessarily receive the full course of therapy because of missed or adjusted doses. Moreover, the patients’ body surface area taken from the lorlatinib clinical^14^ trial and wastage were considered in the analysis, to allow for the fact that any unused vials will be disposed of and not reused with another patient.

The reimbursed drug costs of lorlatinib and other comparators were calculated on the grounds of the ex-factory prices, as they were published in the bulletin issued by Greek Ministry of Health,^23^ after applying the relevant discounts as described in the corresponding legislation at the time of the model development (Table 2).

**Table 2.** Overview of Cost Inputs Considered in the Model.

**Pre-progression Costs**					
**Treatment**	**Dosing Regimen**	**Strength (mg)**	**No. of Vials/Tablets**	**Ex-Factory Price**	**Source**
Lorlatinib	100 mg/day	100	30	€5757	Drug price bulletin, Greek Ministry of Health^23^
Pemetrexed	500 mg /m^2^ every 3 weeks	500	1	€545	
		100	1	€108	
Carboplatin	750 mg every 3 weeks	150	1	€17.23	
		450	1	€48.79	
Cisplatin	75 mg /m^2^ every 3 weeks	10	1	€4.14	
		50	1	€12.59	
		100	1	€24.89	
**Administration Costs per Cycle**		**Unit Cost**		**Source**	
Lorlatinib		€0			
P-ChT		€112.20		Government gazette (Law 2150/27-9-2011) and drug price bulletin (Greek Ministry of Health)^23^	
**Monitoring cost per cycle**		€57.69		EOPYY website^24^ and government gazette (Law, B′1181/8, May 2014)	
**Post-progression Costs**		**Unit Cost**			
Monitoring cost per cycle		€57.69		EOPYY website^24^ and government gazette (Law, B′1181/8, May 2014)	
Subsequent treatment cost per patient		€245.49		Drug price bulletin, Greek Ministry of Health^23^	
End-of-life care cost per patient		€7665		Kokkotou et al^25^	

Abbreviation: P-ChT, pemetrexed plus carboplatin or cisplatin.

The reimbursed drug cost of the combination of pemetrexed plus carboplatin/cisplatin was weighted based on the proportion of split of carboplatin and cisplatin taken from PROFILE 1014,^26^ as this represents the closest population to the decision problem for which a breakdown of platinum-based agents was reported.^26^ Therefore, the model considered that 46.15% of patients receive carboplatin and the remaining 53.85% receive cisplatin.

Additionally, the drug administration cost for intravenous treatments was considered only in the case of chemotherapy infusions. The administration cost (Law 2750/27-09-2011) and premedication cost were calculated for each visit (€112/ visit). Because lorlatinib is self-administered as a tablet, it was assumed that it does not accrue any administration costs (Table 2).

Patient monitoring during treatment includes laboratory tests, diagnostic tests and physician visits, and related unit costs (**Supplementary Table 2**) obtained from the government gazette (Law B′1181/8 May 2014) and the official website of the National Organization for Healthcare Services Provision (EOPYY).^24^ Monitoring costs were health state–specific and not treatment-specific (Table 2).

The management cost of AE grade ≥3 depends on the setting in which these are treated (ie, inpatient or outpatient). Costs for treating and managing AE in the inpatient setting were obtained from the corresponding Diagnostic Related Group tariffs^27^ issued by the Greek Ministry of Health. Costs related to the management of AEs in an outpatient setting were estimated by combining the resources consumed with the corresponding unit costs obtained from the drug price bulletin issued by Greek Ministry of Health,^23^ the government gazette (Law, B′1181/8 May 2014), and the official website of EOPYY.^24^ Finally, a weighted cost for the management of AEs was calculated (**Supplementary Table 3**).

**Post-progression state costs:** Similar to the pre-progression state costs, monitoring costs in the post-progression state consisted of the number of physician visits, diagnostic and laboratory tests, the related unit costs (**Supplementary Table 2**) obtained from the government gazette (Law B′1181/8 May 2014), and the official website of EOPYY.^24^ Post-progression monitoring cost per cycle is reported in Table 2.

Subsequent treatments following progression and cessation of initial treatment are included in the model and are applied at the point of progression; these are assumed to affect only costs. The proportion of patients receiving subsequent treatments in each cycle and generating the according cost was estimated in the model as the proportion of patients who transition from pre-progression to the post-progression health state without dying. This was estimated using the proportion of progression events which were deaths from the lorlatinib clinical trial^14^ and applying this proportion to the proportion of patients leaving the progression-free health state in each cycle.

More specifically, in the base case analysis all patients after lorlatinib and P-ChT were assumed to receive docetaxel. The total costs of subsequent treatment include the drug acquisition costs and administration costs of docetaxel (Table 2).

The end-of-life care cost was directly extracted from a recent Greek retrospective study.^25^ A one-time terminal care cost of €7655 was considered in the model, including expenses for supporting patients during terminal stage by providing them the comfort needed (Table 2). This was applied on transition to the death health state.

### Sensitivity Analyses

To assess the impact of the assumptions considered in the base case analysis, one-way sensitivity analysis (OWSA) was undertaken to test the robustness of the results by varying individual parameters between low and high values. If reported in the literature, confidence intervals (95%) were used in the OWSA; otherwise, if the parameter uncertainty was unknown, a standard 20% variation was used. The tornado diagrams present the 10 parameters with the biggest impact on base case model results.

Moreover, probabilistic sensitivity analysis (PSA) assesses the stochastic parametric uncertainty, which provides an estimate of the joint uncertainty of costs and effectiveness in the simulation, by assigning probabilistic distributions to key input parameters, recursively resampling new values for each parameter from their respective distribution, and subsequently estimating the costs and effectiveness of each intervention based on the new values. This process is repeated multiple times (5000 individual simulations) to provide an estimate of the uncertainty surrounding the cost-effectiveness of interventions. Appropriate distributions were used, specifically, beta distributions for parameters bounded between 0 and 1, log-normal distributions for HR, multivariate normal distributions for correlated parameters such as survival curve parameters, Dirichlet distribution for multinomial distributions, and normal distributions for all other parameters.

## Results

### Deterministic Results

The analysis showed that, over a lifetime horizon, the total costs for lorlatinib and P-ChT arms were estimated to be €81 754 and €12 343, respectively. This difference was attributed to the acquisition cost of lorlatinib compared with P-ChT (€69 039/ patient). However, lorlatinib was more effective than P-ChT with 2.4 and 1.5 more LYs and QALYs gained, respectively. The generated ICERs of lorlatinib compared with P-ChT were €28 613 per LY gained and €46 102 per QALY gained (Table 3).

**Table 3.** Cost-effectiveness Results.

Breakdown Costs	Lorlatinib	P-ChT	Incremental
Drug costs	€72 157	€3118	€69 039
Administration costs	€0	€390	-€390
Monitoring costs	€1803	€294	€1509
Adverse event costs	€24	€91	-€67
Subsequent therapy costs	€602	€629	-€27
Terminal care costs	€7168	€7821	-€653
Total costs	€81 754	€12 343	€69 411
QALYs	1.79	0.28	1.50
LYs	2.85	0.42	2.42
ICER per QALY gained			€46 102
ICER per LY gained			€28 613

Abbreviations: ICER, incremental cost-effectiveness ratio; LY, life year; P-ChT, pemetrexed plus carboplatin or cisplatin; QALY, quality-adjusted life year.

### One-Way Sensitivity Analysis

**Figure 2.**
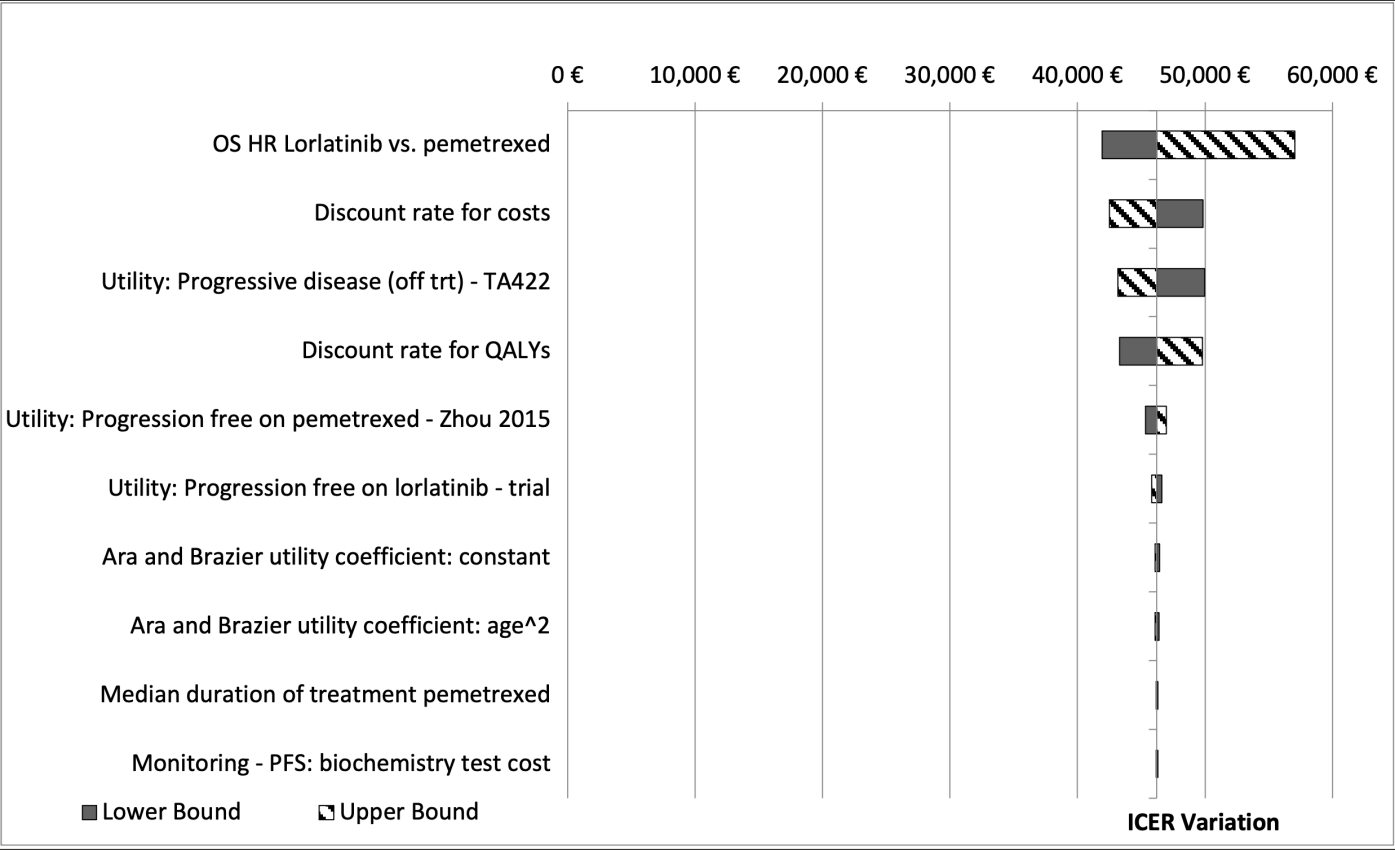
Tornado Diagram of One-Way Sensitivity Analysis: Loratinib vs P-ChT.

An OWSA series was conducted to test how model outputs changed when one parameter at a time was altered. The OWSA results are robust with respect to changes in parameter inputs that are clinically reasonable and are most sensitive to changes in HR of lorlatinib vs P-ChT and discount rate for cost (Figure 2).

### Probabilistic Sensitivity Analysis

**Figure 3.**
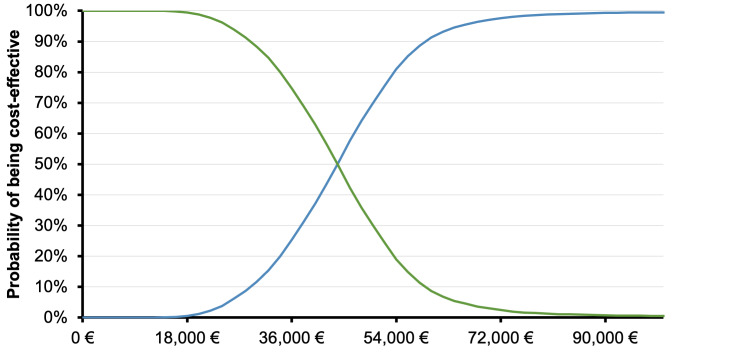
Cost-effectiveness Acceptability Curve: Loratinib vs P-ChT.

The PSA confirmed the base case results, since the likelihood of lorlatinib being cost-effective was higher than 75% compared with P-ChT, at a willingness-to-pay (WTP) equal to €54 000 (3 times the gross domestic product [GDP] per capita of Greece)(Figure 3).^28^ The cost-effectiveness scatterplot, depicting the incremental results between lorlatinib and P-ChT by replication, is generally in line with the base case results, suggesting differences in terms of cost and outcomes between the comparators.

## Discussion

In the present study, an economic evaluation analysis was undertaken from a Greek payer perspective to examine the long-term cost-effectiveness of lorlatinib compared with P-ChT in patients with ALK-positive advanced NSCLC previously treated with ≥1 ALK TKIs in Greece.

Over a lifetime horizon, the model showed that lorlatinib is associated with greater improvements in life expectancy and quality-adjusted life expectancy than P-ChT, with higher associated direct medical costs, generating ICERs of €46 102 per QALY gained and €28 613 per LY gained vs P-ChT. Sensitivity analyses indicated that the base case results were relatively insensitive to variation in input parameters and assumptions. Furthermore, the stability of base case findings was further endorsed by PSA results, which revealed that the probability of lorlatinib being a cost-effective option vs P-ChT was 78%, at the predetermined threshold of €54 000 per QALY gained.

While currently there is no officially established WTP threshold in Greece, lorlatinib can be considered a cost-effective treatment option under the predetermined threshold of €54 000 per QALY gained. This assumption is based on published studies which recommend that, in absence of a local WTP threshold, a health intervention should be considered as cost-effective for a specific country if the generated ICER is between 1 and 3 times the GDP per capita of that country.^29–31^ Furthermore, a recent study^28^ systematically reviewed the WTP threshold used in Greek cost-effectiveness studies over the last 10 years. The study results indicated that, in alignment with other countries, where there is no standard WTP threshold to promote efficient use of healthcare resources, the most prominent practice in Greece was found to be that of 1-3 times the GDP per capita, irrespective of type of treatment or outcome studied. This also aligns with the findings presented in the international literature.^29–31^

Until the time of writing, few published studies have evaluated the cost-effectiveness of lorlatinib compared with alternative therapies in the treatment of patients with advanced, ALK-positive NSCLC. However, it is important to mention that our findings are in line with a recently published study conducted in Sweden.^15^ More specifically, study results suggest that second-line or later treatment with lorlatinib is likely to be a cost-effective option for patients with ALK-positive NSCLC.^15^ Furthermore, it should be highlighted that the calculated ICERs in the present study are lower compared to ICERs calculated in other economic evaluation studies^32,33^ for innovative NSCLC treatments carried out in Greece. In particular, the reported ICERs of these published studies ranged from €85 000^33^ and close to €100 000^32^ per QALY gained, indicating the diversity of ICERs produced in oncology cost-effectiveness models, based on comparative differences in price and outcomes.

Due to the nature of economic models, the present model has some limitations. First, there was a lack of direct clinical evidence for the comparison of treatments in ALK NSCLC. More specifically, the lorlatinib phase II clinical trial was a noncomparative, single-arm trial; as such, there was no common network of comparison for lorlatinib and chemotherapy. Thus, standard indirect treatment comparisons or network meta-analyses were not possible. MAIC^15^ was used, as it allows a comparison for chemotherapy against lorlatinib. This methodology requires information for the comparator regarding patient characteristics for treatment effect modifiers and prognostic factors for the disease. It was assumed that the HR estimated from the MAIC based on the EXP2-3A cohorts was applicable to the cohort of interest, thus assuming that the relative treatment effect is constant irrespective of the population. Further, the use of an HR assumes that the hazards are proportional over time. Both assumptions were necessary in the absence of further relevant information. Nonetheless, the sensitivity analyses showed that the model results were robust to changes in the key parameters unless the HR values approached unrealistic levels. In addition, these clinical data were also considered in a recent publication of the model.^15^ Second, the present analysis assumed that the utility data obtained from published studies were applicable to the Greek healthcare setting. The use of these data may be questioned; however, given the absence of local data and the quality and validity of the respective studies, this choice was considered appropriate. Despite the uncertainty around these assumptions, a series of sensitivity analyses indicated that model outcomes are robust, since the main results remained unchanged in a wide range of parameter values. Finally, it should be noted that the results must be strictly considered within the Greek setting and on the basis of the present resource and drug prices. If any of the underlying parameters change, so may the results and the conclusions of the analysis.

## Conclusion

Based on available clinical, local resource utilization, and unit cost data, the present economic evaluation suggests that lorlatinib, a potent, brain-penetrant, targeted therapy covering the unmet medical need of patients with advanced, ALK-positive NSCLC who have progressed after treatment with at least one second-generation TKI, was found to be a cost-effective option over P-ChT in Greece.

### Disclosures

GG received consulting fees from Pfizer Hellas. OZ, NB, and CM are Pfizer employees. CL is an employee of BresMed. BresMed received consultancy fees from Pfizer Inc for the development of the global cost-effectiveness model for lorlatinib and supporting statistical analyses. The authors did not receive direct payment as a result of this work outside their normal salary payments. None of the other authors has any personal or financial conflict of interest.

## Supplementary Material

Supplementary Online Material
