# Supplementary material for: Cost-effectiveness Analysis of Lorlatinib in Patients Previously Treated with Anaplastic Lymphoma Kinase Inhibitors for Non-small Cell Lung Cancer in Greece
Source: J Health Econ Outcomes Res. 2022 Feb 17;9(1):50–7. doi: 10.36469/jheor.2022.32983 (PMC8853822; doi:10.36469/jheor.2022.32983)
Supplement: Supplementary Online Material [file jheor_2022_9_1_31983_82012.pdf]

Journal of Health Economics and Outcomes Research

Online Supplementary Material

Cost-effectiveness Analysis of Lorlatinib in Patients Previously Treated with Anaplastic Lymphoma Kinase Inhibitors for Non-small Cell Lung Cancer in Greece. *JHEOR*. 2022;9(1):50-57. doi:10.36469/jheor.2022.32983

Table S1: Frequencies of Adverse Events Considered in the Model

Table S2: Monitoring Cost in Pre- and Post-progression State

Table S3: Adverse Event Management Cost Considered in the Model

This supplementary material has been provided by the authors to give readers additional information about their work.

This is an open-access article distributed under the terms of the Creative Commons Attribution 4.0 International License (CCBY-4.0). View this license's legal deed at http://creativecommons.org/licenses/by/4.0 and legal code at http://creativecommons.org/licenses/by/4.0/legalcode for more information.

Table S1: Frequencies of Adverse Events Considered in the Model

Adverse Event	Lorlatinib[a]	P-ChT[b]
Increased alanine aminotransferase	0%	1.4%
Increased aspartate aminotransferase	0.4%	0.7%
Diarrhea	0.4%	0.7%
Fatigue	0.4%	0%
Hypercholesterolemia	15.6%	0%
Hypertriglyceridemia	15.6%	0%
Nausea	0%	2%
Anemia	0%	1.4%
Neutropenia	0%	14.3%

Abbreviation: P-ChT, pemetrexed plus carboplatin or cisplatin.
[a] Source: Solomon et al.[14]
[b] Sources: Novello et al[7]; Shaw et al.[20]

Table S2: Monitoring Cost in Pre- and Post-progression State

Resource Item	Cost/Test (€)	% of Patients	No. of Tests/ Month
**Health professional visit**			
Oncologist	10.00	80	1
**Laboratory tests**			
Complete blood count	1.69	80	2
Chest x-ray	3.44	30	1
Biochemical analysis	22.07	80	2
CT scan (chest)	38.25	30	1
**Total cost per cycle in pre- and post-progression**			**€57.69**

Table S3: Adverse Event Management Cost Considered in the Model

Adverse Event	Unit Cost/ Event (€)	Source
Increased alanine aminotransferase	141.66	DRGs tariffs,[26] EOPYY website,[25] government gazette (Law, B′1181/8 May 2014), and the drug price bulletin[23] .
Increased aspartate aminotransferase	145.60	
Diarrhea	73.58	
Fatigue	46.44	
Hypercholesterolemia	79.98	
Hypertriglyceridemia	77.95	
Nausea	57.63	
Anemia	833.83	
Neutropenia	527.63	

Abbreviation: DRG, Diagnostic Related Group.
